# Ethanolic Extract of Pomegranate (*Punica granatum* L.) Prevents Oxidative Stress and Preserves the Morphology of Preantral Follicles Included in Bovine Ovarian Tissue Cultured In Vitro

**DOI:** 10.3390/ani15223344

**Published:** 2025-11-19

**Authors:** Maria Alice Felipe Oliveira, Solano Dantas Martins, Ernando Igo Teixeira de Assis, Jonathan Elias Rodrigues Martins, Fernanda Lima Alves, Sara Rany Alexandre Bittencourt, Ingrid Gracielle Martins da Silva, Sônia Nair Báo, Queli Cristina Fidelis, Selene Maia de Morais, José Roberto Viana Silva, Vânia Marilande Ceccatto, Valdevane Rocha Araújo

**Affiliations:** 1 Graduate Program in Biotechnology (PPGB), Federal University of Ceará (UFC), 100, Comandante Maurocélio Rocha Ponte Avenue, Sobral 62041-040, CE, Brazil; alicemafo2@gmail.com (M.A.F.O.); solanodantas59@gmail.com (S.D.M.); jrvsilva@ufc.br (J.R.V.S.); 2 Laboratory of Research in Reproductive Physiology (FisioRep Lab), Parnaíba Delta Federal University, 2819, São Sebastião Avenue, Parnaíba 64202-020, PI, Brazil; fe.lima.alves@gmail.com (F.L.A.); sarabittencourtsrb@gmail.com (S.R.A.B.); 3 Laboratory of Biotechnology and Physiology of Reproduction—LABIREP, Biotechnology Nucleus of Sobral—NUBIS, Federal University of Ceará (UFC), 100, Comandante Maurocélio Rocha Ponte Avenue, Sobral 62041-040, CE, Brazil; ernandoigor@gmail.com; 4 Laboratory of Biochemistry and Gene Expression (LABIEX), Higher Institute of Biomedical Sciences (ISCB), State University of Ceará (UECE), 1700, Av. Dr. Silas Munguba, Fortaleza 60714-903, CE, Brazilvania.ceccatto@uece.br (V.M.C.); 5 Microscopy and Microanalysis Laboratory, Department of Cell Biology, Institute of Biological Sciences, University of Brasília (UnB), s/n, Brasília 70910-900, DF, Brazil; ingridgracielle@unb.br (I.G.M.d.S.); snbao@unb.br (S.N.B.); 6 Laboratory of Natural Products Chemistry, Federal University of Maranhão (UFMA), 1966, Portugueses Avenue, São Luiz 65080-850, MA, Brazil; qc.fidelis@ufma.br; 7 Postgraduate Program in Natural Sciences (PPGCN), State University of Ceará (UECE), 1700, Av. Dr. Silas Munguba, Fortaleza 60714-903, CE, Brazil; selenemaiademorais@gmail.com

**Keywords:** in situ culture, redox balance, antioxidants, plant extract, punicalagin

## Abstract

Oxidative stress compromises follicle survival during in vitro culture. Natural antioxidants may reduce cellular damage and preserve tissue integrity. This study evaluated the ethanolic extract from *Punica granatum* L. (EE-PG) on bovine ovarian tissue cultured in vitro. Bovine ovarian (*n* = 24) fragments were cultured for 6 days in αMEM^+^ medium with or without EE-PG at 10, 50, or 100 µg/mL. At the end of the in vitro culture, the medium was used to evaluate antioxidant capacity (DPPH and ABTS assays), while the fragments were collected for morphological and biochemical analyses. HPLC-UV-Vis confirmed the presence of α-punicalagin in the pure EE-PG. At 100 µg/mL, EE-PG showed the strongest effects: it had higher antioxidant capacity, preserved follicle morphology and ultrastructure, and promoted follicle activation. At 50 and 100 µg/mL, the extract also reduced malondialdehyde (MDA) and increased thiol levels, indicating protection against lipid peroxidation. In contrast, 10 µg/mL had little effect. Follicle and oocyte diameters were not significantly altered, but the collagen I/III ratio increased at higher concentrations, suggesting extracellular matrix remodeling. Together, these findings demonstrate that EE-PG protects bovine preantral follicles from oxidative stress, maintains redox balance, and preserves tissue integrity. These results reinforce the potential of *Punica granatum* L. extract as a natural antioxidant in reproductive biotechnologies and fertility preservation.

## Simple Summary

Oxidative stress is a major challenge for animal reproduction, especially in cattle (bovines), and is caused by diseases like mastitis, which intensify cellular ‘rusting’ (cellular damage/oxidation). This process acts similarly to the rust that damages cars; within cells, oxidative stress destroys small structures that protect the immature eggs, known as ovarian follicles, preventing them from growing and maturing correctly. To protect these delicate structures, it is necessary to find a substance that can prevent this attack—that is, an antioxidant. For this reason, we focused our attention on the pomegranate (*Punica granatum* L.), a fruit known to be rich in natural antioxidants. We discovered that its extract is effective: it acted as a protective shield, maintaining the health of the bovine follicles and reducing cellular ‘rusting,’ while simultaneously restoring their internal balance (the redox balance). We demonstrated that pomegranate can be used as a natural and powerful additive to protect and maintain the quality of bovine ovarian tissue in the laboratory—a crucial finding for improving assisted reproductive techniques in livestock.

## 1. Introduction

Folliculogenesis in mammals is a complex physiological process, and the mechanisms responsible for its regulation are not yet fully understood. Consequently, many studies have been performed using reproductive biotechnologies to facilitate the investigation of the mechanisms underlying folliculogenesis. These biotechnologies allow in vitro growth and development of the follicles and oocytes, and even embryo production. Moreover, these techniques are essential for human reproductive health and animal production since they can improve female reproductive capacity and support fertility preservation programs [1]. However, the gametes may suffer damage due to oxidative stress induced by in vitro conditions, such as variations in temperature and oxygen levels, as well as excessive light exposure [2].

Oxidative stress causes cellular damage due to redox imbalance, which occurs when there is a disproportion between the production of free radicals and endogenous antioxidants. This defense system is mediated by enzymes such as superoxide dismutase (SOD), catalase, and glutathione. Redox imbalance leads to the oxidation of critical cellular structures, including proteins, lipids, and DNA [3]. Consequently, several studies have investigated substances capable of mitigating the deleterious effects of oxidative stress during in vitro culture [4]. Recent studies on antioxidants have shown that these substances directly affect sperm quality and pregnancy rates in humans, suggesting that they may play an important role in fertility [5]. In this context, various plants and their extracts have become frequent targets of scientific investigation. Among these, pomegranate (*Punica granatum* L.), widely known in traditional medicine, stands out as a promising natural source of bioactive compounds.

Among the compounds found in pomegranate, punicalagin and ellagic acid are the most abundant in the fruit. These substances are phenolic compounds [6], which are responsible for most of pomegranate benefits, such as antioxidant [7,8,9] and antitumor activities [7] in various tissues already studied. Previous studies have reported that ellagic acid possibly regulates cellular pathways involved in growth and apoptosis, since it inhibits ovarian cancer cells’ proliferation and migration [10,11]. As for punicalagin, our team has demonstrated that it improves follicle development and promotes antioxidant enzymes activities [12]. Moreover, the combination of these constituents has been shown to exhibit synergistic effects [13,14], which make us believe that the pomegranate extract may be powerful, including in reproductive tissues.

Solutions derived from the peel or seeds of *Punica granatum* fruits have demonstrated a range of effects, particularly antioxidant properties. It was observed that pomegranate seed oil reduces oxidative stress and NADPH oxidase activity, as well as TNF-α levels, while promoting increased antioxidant activity in ovarian tissue subjected to ischemia and reperfusion [15]. Although studies have demonstrated the antioxidant effects of pomegranate, there is a scarcity of research evaluating the effects of this plant on antioxidant pathways in bovine ovarian tissue cultured in vitro. Thus, the present study aimed to investigate whether the ethanolic extract of *Punica granatum* (EE-PG) could preserve follicle morphology and maintain redox balance in cultured bovine ovarian tissue. By addressing this gap, we also discuss the potential implications of EE-PG for reproductive biology and fertility preservation.

## 2. Materials and Methods

### 2.1. Chemicals and Ethical Approval

Unless mentioned otherwise, the Chemical reagents were purchased from Sigma-Aldrich (St. Louis, MO, USA). This study was approved by the Ethics Committee for Use of Animals from State University of Ceará (UECE) (CEUA/UECE no. 09326942/2021).

### 2.2. Production of the Ethanolic Extract from the Fruit Peel of Punica granatum L. and Chemical Analyses

The fruit peel of *Punica granatum* L. was obtained from fresh and ripe fruits sourced from commercial cultivation. The peels were removed from the fruit and dried at room temperature. After drying, the peels (250 g) were pulverized using an electric grinder. Extraction was performed by maceration using ethanol (PA 99.8%) as a solvent, for 5 days. Afterward, the extractive solution was filtered through a glass funnel with filter paper to remove fibers and impurities. The resulting liquid was concentrated in a rotary evaporator at 50 °C, where the solvent was completely evaporated, leaving only the concentrated extract for subsequent analyses [16]. Finally, this extract was lyophilized and stored at a temperature equal to or below 10 °C. The powder, called ethanolic extract from *Punica granatum* (EE-PG, 1 g), was dissolved in ultrapure water to be used in experiments at concentrations of 10, 50, and 100 µg/mL [7]. Access to this genetic resource was registered on the SISGEN platform, no. A46E154.

### 2.3. High-Performance Liquid Chromatography (HPLC-UV-Vis)

The sample was dissolved in spectroscopic-grade methanol to obtain a concentration of 2 or 3 mg/mL. Subsequently, 20 µL of each sample was injected into a Shimadzu high-performance liquid chromatography (HPLC-UV-Vis) (São Paulo, Brasil) system with a UV-Vis detector, using a Luna C18 column (150 mm × 4.8 mm × 5 µm) and a wavelength of 254 nm. The mobile phase consisted of solvent A (water/acetic acid—98:2) and solvent B (methanol), with an elution gradient of: 0.1–10 min, 10% of B; 10–15 min, 50% of B; 15–20 min, 70% of B; 20–30 min, 80% of B; 30–40 min, 50% of B; 40 min, 5% of B [17].

### 2.4. Source of Ovaries and In Vitro Culture of Ovarian Tissue

Ovaries (*n* = 24) from adult cows of undefined breed were collected from a local slaughterhouse and transported to the laboratory within 2 h in physiological saline (0.9% NaCl) supplemented with penicillin (100 µg/mL) and streptomycin (100 µg/mL) at 4 °C. Each pair of ovaries was divided into 3 × 3 × 1 mm fragments isolated from the cortex, which were randomly placed in a 24-well plate with 500 µL (2 fragments/well) of minimum essential medium alpha modification (αMEM) base medium (M1142; Vitrocell Embriolife, São Paulo, Brazil) supplemented with ITS (10 µg/mL insulin, 5.5 µg/mL transferrin, and 5 ng/mL selenium; I3146 Sigma-Aldrich; St. Louis, MO, USA), 2 mM hypoxanthine (H9636 Sigma-Aldrich; St. Louis, MO, USA), 2 mM glutamine, and 1.25 mg/mL Bovine Serum Albumin (BSA; A9647 Sigma-Aldrich; St. Louis, MO, USA), and was called αMEM^+^. The medium was pre-incubated for at least 2 h [18].

The cultures were maintained for 6 days [18] at 38.5 °C in a humidified atmosphere with 5% CO_2_, with different concentrations (10, 50, and 100 µg/mL) of the ethanolic extract from *Punica granatum* L. (EE-PG). Partial medium changes were performed every 48 h. At the end of the culture period, the tissues were either fixed or frozen for subsequent morphological (*n* = 10 Ovaries) and biochemical analyses (*n* = 14 Ovaries), respectively. The 6-day culture period was defined based on previous studies as the minimum time required to obtain follicular activation (transition from primordial to primary follicle) [19]. Moreover, longer periods, such as 16 days in this culture system, are frequently associated with decreased viability and follicular degeneration [20].

### 2.5. Evaluation of Total Antioxidant Capacity by the DPPH Method

On days 2 and 6 of in vitro culture, the antioxidant capacity of the culture medium was assessed using the stable radical DPPH, following established protocols with modifications. A stock solution was prepared by dissolving 13.8 mg of DPPH in 100 mL of methanol and stored under refrigeration until use. For the assay, 20 μL of culture medium were mixed with 200 μL of the methanolic DPPH solution and incubated for 60 min in the dark at 25 °C. Methanol (20 μL) served as the blank, and additional controls were performed by adding DPPH directly to the medium to correct for sample color interference. Absorbance was recorded at 490 nm using a Biotek ELISA microplate reader (model ELX 800 with Gen5 V2.04.11 software; São Paulo, Brasil). All reactions were carried out in triplicate. Serial dilutions of the samples (ranging from 2000 to 0.78 μg/mL) were prepared, and antioxidant activity was expressed as the percentage of radical inhibition according to the formula:

$$AA\%=\;\frac{AC-AS\;}{AC}\;\times 100$$

where *AA* (%) stands for the percentage of antioxidant activity, *AC* stands for the absorbance of the negative control, and *AS* stands for the absorbance of the sample. The mean inhibitory concentration (IC50; μg/mL) was obtained using calibration curves, collected by plotting the different concentrations in relation to *AA*%, and further analyzed by linear regression [21].

### 2.6. Evaluation of Total Antioxidant Capacity by the ABTS Method

The antioxidant activity of the culture medium was also determined by the ABTS assay on days 2 and 6 of culture, with adaptations from previously described protocols [21]. A stock solution of ABTS (7 mM, 5 mL) (Sigma-Aldrich; St. Louis, MO, USA) was mixed with 88 μL of potassium persulfate (140 mM) and incubated for 16 h in the dark at room temperature to generate the ABTS^+^ radical cation. Afterwards, 1 mL of this mixture was diluted with 99 mL of ethanol. For the assay, 30 μL of each sample dilution were added to 3 mL of the ABTS^+^ solution, and absorbance was measured at 630 nm after 6 min of reaction. The antioxidant activity (*AA*%) and IC50 values were determined using the same approach as described for the DPPH assay [21].

### 2.7. Morphological and Follicular Development Analysis by Histology

Immediately after ovary collection (fresh control, D0), as well as at the end of the culture period (D6), ovarian fragments (*n* = 3 fragments) from each treatment were fixed in paraformaldehyde for 12 h at 4 °C. Then, fragments were dehydrated in increasing concentrations of ethanol, following standardized protocol for histological analysis. Sections of 7 µm were made and subsequently stained with hematoxylin-eosin (HE). Follicles were classified as primordial (one layer of flattened granulosa cells around the oocyte) or growing follicle, i.e., primary (a single layer of cuboidal granulosa cells around the oocyte), or secondary (oocyte surrounded by two or more layers of cuboidal granulosa cells), as described by [22]. These follicles were classified individually as histologically normal when an intact oocyte was present, i.e., an oocyte without a pyknotic nucleus or cytoplasmic retraction surrounded by granulosa cells well organized in one or more layers and have no pyknotic nucleus. Atretic follicles were defined as those with a retracted oocyte, pyknotic nucleus, and/or disorganized granulosa cells detached from the basement membrane.

### 2.8. Ovarian Stromal Cell Density in In Vitro Cultured Ovarian Tissue

The evaluation of stromal cell density was performed in fresh control and in tissues cultured for 6 days in the different treatments using HE slides. Six slides from each animal and from each treatment were evaluated with the aid of a camera attached to a microscope, totaling 30 slides per treatment. From each slide, four random fields (50 × 50 µm  =  2500 µm^2^) were recorded to calculate the mean stromal cell density using the DS cooled camera head DS196 Ri1 coupled to a microscope (Nikon Eclipse 80i, Tokyo, Japan; 400× magnification). All evaluations and measurements were performed by a single operator [22].

### 2.9. Analysis of the Extracellular Matrix (ECM) in In Vitro Cultured Ovarian Tissue

Collagen fibers in extracellular matrix of the ovarian cortex were stained with Picrosirius Red (Abcam Kit; Nova Analítica, São Paulo, Brasil), as previously described in [23,24] with modifications. After standard histological process, as previously described, ovarian sections of 7 μm were dewaxed in xylene and incubated in Sirius Red solution (0.1%) for 1 h at room temperature. Then, the excess dye was removed with acetic acid solution (0.5%) and the sections were dehydrated and subjected to slide assembly with subsequent observation under a polarizing microscopy (400× of magnification, Nikon E200; Tokyo, Japan) coupled to an image capture system (Nikon, Coolpix 4500; Tokyo, Japan). The total collagen fiber content in the connective tissue and the different types of collagen fibers based on polarizing colors (i.e., collagen type I fibers stained yellow with orange birefringence and collagen type III fibers stained with green birefringence) were analyzed as previously described [25]. Fifty sections were evaluated per treatment. Images were analyzed by red, green, and blue threshold measurement to obtain the percentage of red and green colors (expressed in pixels) and 3D data representation of the pixels for each image was performed using ImageJ software (version 1.54k). The blue color representing all the other cellular types was omitted.

### 2.10. Evaluation of Ultrastructural Morphology of Bovine Preantral Follicles After in Culture by Transmission Electron Microscope

For evaluation of the ultrastructural follicular morphology, only fragments were used from fresh control, αMEM alone, as a control of the in vitro environment, and 100 µg/mL of EE-PG, as the treatment choose after histological analysis. Small pieces (1 mm^3^) of ovarian tissues were fixed in 2% paraformaldehyde and 2.0% of glutaraldehyde in 0.1 M of sodium cacodylate buffer (pH 7.2) for 24 h at room temperature. After fixation, fragments were washed with 0.1 M of sodium cacodylate buffer. Subsequently, samples were post-fixed for 1 h in osmium tetroxide 2% and potassium ferricynide 1.6% in sodium cacodylate buffer 0.2 M, and then contrast was done in bloc in an aqueous solution of uranyl acetate 0.5% for 2 h. Dehydrated through a gradient of acetone (30–100%) and the tissues were embedded in Spurr resin. Semi-thin sections (3 µm) were cut on an ultramicrotome (Leica EM UC7, Vienna, Austria) for light microscopy studies and stained with toluidine blue. Uranyl acetate and lead citrate were used to stain the ultrathin sections (60–70 nm) for contrast, and sections were examined under a transmission electron microscope (Jeol 1011, Tokyo, Japan). Parameters, such as density and integrity of the cell organelles, vacuolization, basement membrane and granulosa cells integrity were evaluated [18].

### 2.11. Total Proteins (Bradford Method)

Ovarian fragments (*n* = 14 ovaries) from fresh control and each treatment after 6 days of in vitro culture were frozen to biochemical analysis. The protein concentration was determined using the Bradford method. This method uses Coomassie blue (Quick start/Bradford; Catalogue No. 500–0205; Bio-Rad, Hercules, CA, USA) to determine the total concentration of proteins in each extract sample. When it comes in contact with proteins, the Coomassie blue stain forms a complex and emits a blue luminescence. The absorbance is directly related to the protein concentration of the sample and was evaluated spectrophotometrically at a wavelength of 595 nm. The total protein concentration in samples was determined using a standard curve constructed using bovine albumin as a standard (0, 2.5, 5, 10, 15, 25, 35 and 50 mg/mL) and was used to standardize the levels of pro-oxidants (thiol) and antioxidants (SOD and CAT), as described below.

### 2.12. Evaluation of the Redox Profile Based on Thiol Content

Total thiol content was determined using 5,50-dithiobis 2-nitrobenzoic acid (DTNB; D8130, Sigma-Aldrich, St. Louis, MO, USA) as an index of reduced thiol. Thiol residues react with DTNB (10 mM), cleaving the disulfide bond to form 2-nitro-5-thiobenzoate anion (NTB2^−^) at a neutral pH. NTB2^−^ is quantified in a spectrophotometer by measuring absorbance at 412 nm, with results expressed as nMol of reduced DTNB per milligram of protein [26].

### 2.13. Determination of the Malondialdehyde (MDA) Levels by Production of Thiobarbituric Acid Reactive Substances (TBARS)

The quantification of thiobarbituric acid reactive substances assesses the final product of membrane lipid peroxidation, malondialdehyde (MDA). 63 µL of ovarian tissue homogenate were added to 100 µL of 35% perchloric acid. The samples were centrifuged at 5000 rpm for 10 min at 4 °C. Finally, 150 µL of the supernatant were added to 50 µL of 1.2% thiobarbituric acid then, heated to 95 °C for 30 min. Readings were obtained at A_535_ nm [27].

### 2.14. Determination of Antioxidant Status

For determination of antioxidant enzymes, the ovaries were stored at −80 °C and evaluated as previously described by our team [28].

#### 2.14.1. Activity of the Superoxide Dismutase Activity (SOD) Enzyme

SOD activity was measured from inhibition of adrenaline autoxidation. Adrenaline oxidizes in the presence of a catalase enzyme in basic medium. Therefore, adrenaline oxidation was determined when the catalase enzyme reacted with hydrogen peroxide (H_2_O_2_). The H_2_O_2_ in the medium was generated by the SOD enzyme from the consumption of O_2_^−^ radical, which has a high activity in basic medium [29]. The catalase enzyme solution (0.048 mg/mL, C9322, Sigma-Aldrich Chem. Co, St Louis, MO, USA) was prepared and added to a glycine buffer (7:3, pH 10.2, Dinâmica, São Paulo, Brazil). The ovary homogenate was added and then the adrenaline (0.218 mg/mL, E4260 Sigma-Aldrich Chem. Co, St Louis, MO, USA) to start oxidation. Oxidation was measured at 480 nm every 10 s for 180 s.

#### 2.14.2. Catalase Activity (CAT)

The CAT activity was measured by consumption of H_2_O_2_ as substrate at 240 nm [30]. A quartz bucket was added a solution of H_2_O_2_ (152 μL/mL, PH09717RA Êxodo Científica, São Paulo, Brazil) and phosphate-buffered saline (PBS), pH 7.4, at room temperature, and the ovary protein homogenate proteins were added. Every 30 s, the consumption of H_2_O_2_ was measured twice.

### 2.15. Statistical Analysis

The results are presented as the mean ± standard error of the mean (SEM) of the replicates. Chi-square (Χ^2^) was used to analyze categorical data (follicle normality and activation), and one-way ANOVA was used for biochemical analyses, morphometric measurements (oocyte and follicle diameters), and stromal cell density. Tukey post hoc tests were performed when the normality hypothesis was rejected, as determined by the Shapiro–Wilk test, using GraphPad Prism 7.0 software (GraphPad Software, Inc., San Diego, CA, USA). Statistical significance was set at *p* < 0.05.

## 3. Results

### 3.1. Chemical Composition of the Ethanolic Extract of Punica granatum (EE-PG)

**Figure 1:**
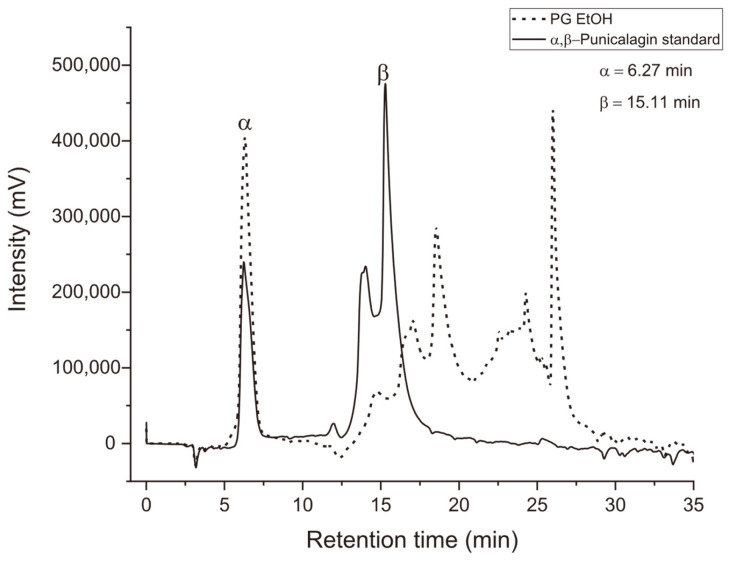
Overlay of HPLC-UV-Vis chromatograms of the ethanolic extract from *Punica granatum* (EE-PG) (dotted line) and the α- and β-punicalagins standards (solid line), in 254 nm.

Chromatographic analysis of EE-PG revealed multiple peaks between 6.29 and 25.97 min of retention time. The peak at 6.29 min matched the retention time of the α-punicalagin standard (6.27 min), confirming its presence in the extract (Figure 1). No peaks corresponding to β-punicalagin were detected.

### 3.2. Effect of EE-PG on Follicular Survival, Development, Follicular and Oocyte Diameters, and Density of Stromal Cells

**Figure 2:**
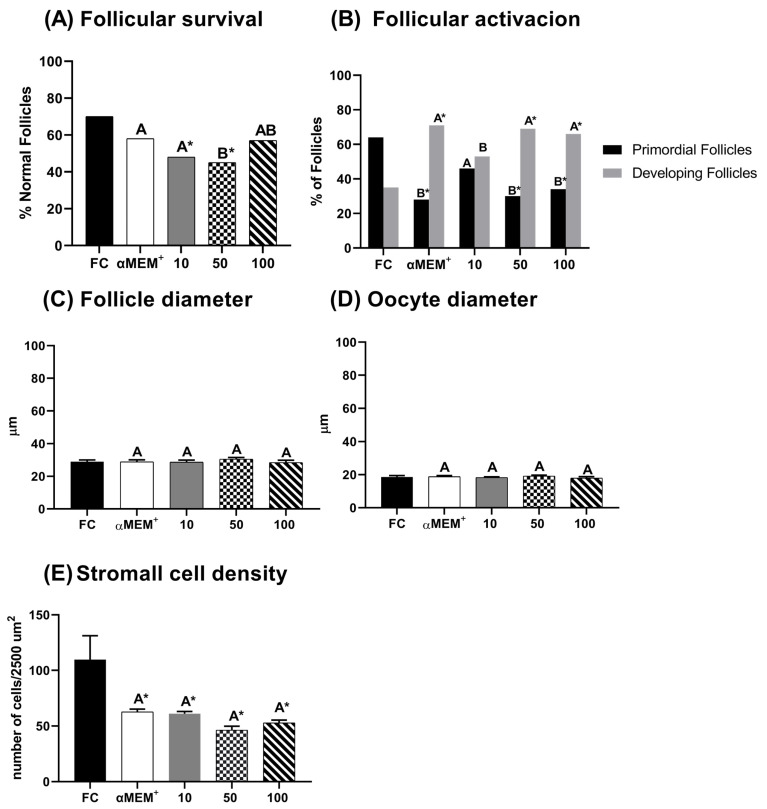
Percentage of morphological normal follicles (**A**), follicular activation (**B**), follicle (**C**) and oocyte (**D**) diameters, and stroma density area (**E**) in bovine ovarian tissue before (fresh control, FC) and after 6 days of in vitro culture in the absence (αMEM^+^) or presence of ethanolic extract from *Punica granatum* L. (EE-PG) at 10, 50 and 100 µg/mL. ^A,B^ Different capital letters denote significant differences among treatments at day 6 of in vitro culture (*p* < 0.05). * Indicates difference from FC (*p* < 0.05). The analyses were performed using the chi-squared test (Χ^2^) (for percentage of normal follicles and follicular activation) and one-way ANOVA followed by Tukey’s post hoc test (for oocyte and follicular diameter and stromal cell density).

Figure 2 demonstrates the percentage of morphologically normal, developing follicles, follicular and oocyte diameters, and stromal cell density. In Figure 2A, low and intermediate concentrations of EE-PG (10 and 50 µg/mL) significantly reduced the proportion of morphologically normal follicles compared to the fresh control (*p* = 0.002 and *p* = 0.0003, respectively). However, only the highest concentration (100 µg/mL) of EE-PG preserved this percentage, being similar to fresh control and αMEM^+^ alone. Figure 2B shows that 50 and 100 µg/mL reduced the percentage of primordial follicles (*p* = 0.0004 and *p* = 0.001, respectively) while increasing the proportion of developing follicles compared to the fresh control. Although no significant difference was observed relative to αMEM^+^. Figure 2E demonstrates that the stromal cell density was significantly reduced in all treatments after in vitro culture when compared to fresh control. Regarding follicular (Figure 2C) and oocyte (Figure 2D) diameters, no statistically significant differences were observed among treatments.

### 3.3. Evaluation of Type I and III Collagen Fibers in the Extracellular Matrix

**Figure 3:**
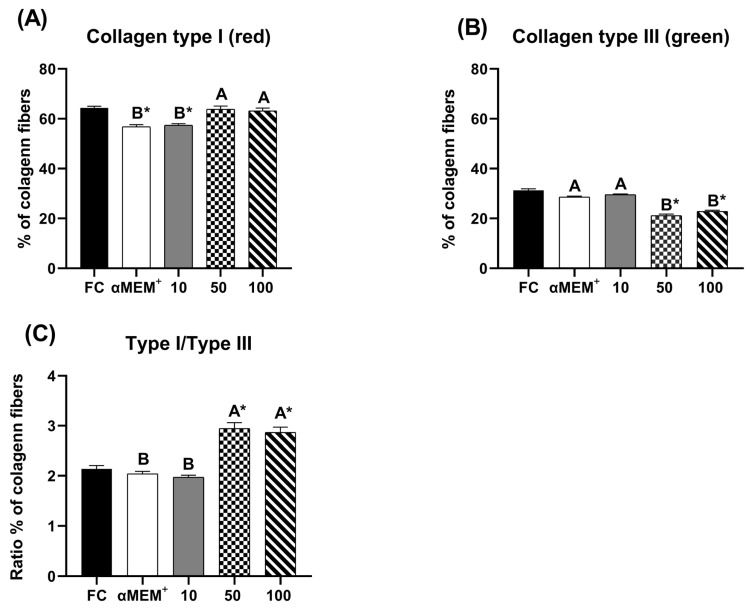
Percentage of pixels for type I (**A**), type III (**B**) and the ratio of type I/type III collagen fibers (**C**) in fresh bovine ovarian tissue (fresh control, FC) or after 6 days of in vitro culture in the absence (αMEM^+^) or presence of ethanolic extract from *Punica granatum* L. (EE-PG) at 10, 50 and 100 µg/mL. ^A,B^ Different capital letters denote significant differences among treatments at day 6 of in vitro culture (*p* < 0.05). * Indicates difference from FC (*p* < 0.05). The analyses were performed using one-way ANOVA followed by Tukey’s post hoc test.

Figure 3 illustrates the distribution of type I and type III collagen fibers, as well as the type I/III collagen ratio. The intermediate and highest concentration of EE-PG significantly increased the proportion of type I collagen (*p* < 0.0001) compared to control cultured (αMEM^+^ alone) and decreased type III collagen (*p* < 0.0001) compared to fresh (D0) and cultured (αMEM^+^ alone) controls. Moreover, the type I/III collagen ratio was higher for these concentrations relative to 10 µg/mL and both controls (*p* < 0.0001).

### 3.4. Effect of EE-PG on Follicular Ultrastructure Preservation

**Figure 4:**
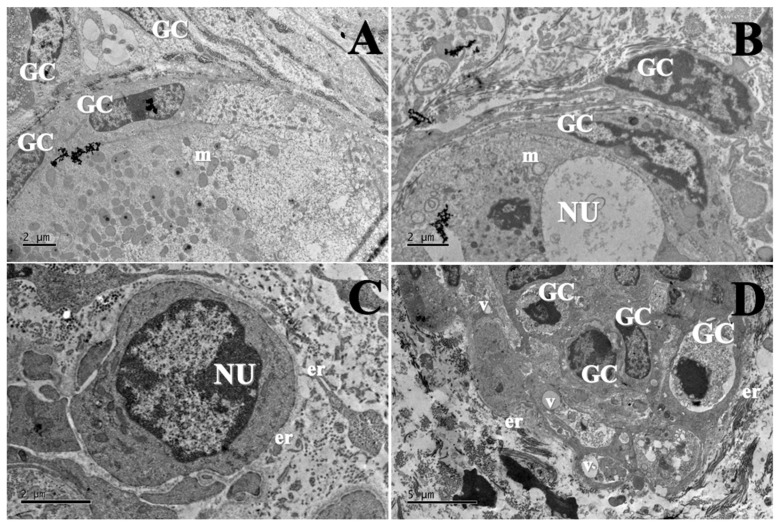
Ultrastructural morphology of preantral follicles from (**A**) fresh control (1500×), (**B**) αMEM^+^ (1500×), (**C**) 10 µg/mL of EE-PG (1200×), and (**D**) 100 µg/mL of EE-PG (1000×). GC: Granulosa cell; NU: Oocyte nucleus; m: mitochondria; er: Endoplasmic reticulum; V: vacuoles.

Morphologically normal follicles were observed only in the fresh control, cultured control and 100 µg/mL EE-PG. Which displayed homogeneous oocyte cytoplasm with numerous mitochondria. In contrast, tissues cultured with 10 µg/mL EE-PG (Figure 4C) showed cytoplasmic retraction, indicative of follicle degeneration.

### 3.5. Total Antioxidant Capacity of EE-PG Assessed by DPPH and ABTS Methods

**Figure 5:**
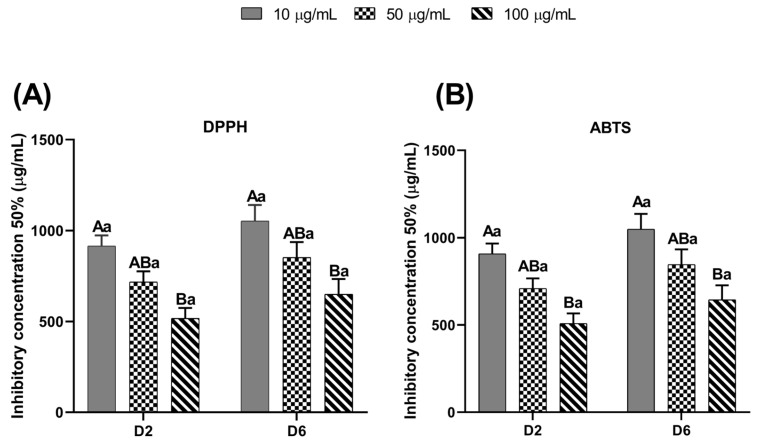
Total antioxidant activity by the DPPH (**A**) and ABTS (**B**) methods of the different concentrations (10, 50 and 100 µg/mL) of ethanolic extract from *Punica granatum* (EE-PG) during in vitro culture (Days 2 and 6) of bovine preantral follicles included in ovarian tissue. ^A,B^ Different capital letters denote significant differences among treatments at the same day of culture (*p* < 0.05). ^a^ Different lowercase letters denote significant differences between days 2 and 6 at the same treatment (*p* < 0.05). The analyses were performed using one-way ANOVA followed by Tukey’s post hoc test.

Figure 5 illustrates the total antioxidant capacity of the EE-PG in the culture medium on days 2 and 6, evaluated by the ability of EE-PG inhibit 50% (IC_50_) of DPPH and ABTS free radicals. Both assays revealed that only highest concentration of EE-PG exhibited significantly greater antioxidant capacity (*p* = 0.0222 and *p* = 0.0173, respectively) when compared to the low concentration, regardless of the culture day.

### 3.6. Effect of EE-PG on Antioxidant Enzyme Activity (SOD and CAT)

**Figure 6:**
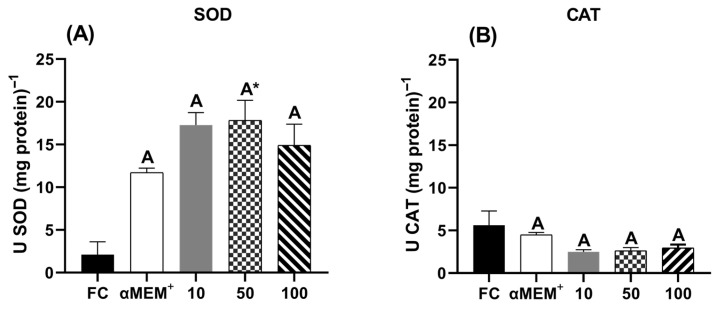
Antioxidant enzyme activity of (**A**) superoxide dismutase (SOD) and (**B**) catalase (CAT) in bovine ovarian tissue before (fresh control, FC) and after 6 days of in vitro culture in the absence (αMEM^+^) or presence of ethanolic extract from *Punica granatum* L. (EE-PG) at 10, 50 and 100 µg/mL. ^A^ Different capital letters denote significant differences among treatments at day 6 of in vitro culture (*p* < 0.05). * Indicates difference from FC (*p* < 0.05). The analyses were performed using one-way ANOVA followed by Tukey’s post hoc test.

Figure 6 shows the activity of the antioxidant enzymes superoxide dismutase (SOD) and catalase (CAT). Notably, only the intermediate concentration (50 µg/mL) significantly increased SOD activity compared to the fresh control (*p* = 0.0312). In contrast, CAT activity showed no statistical differences among treatments.

### 3.7. Redox Profile Evaluation Based on MDA Content and Reduced Thiol Levels

**Figure 7:**
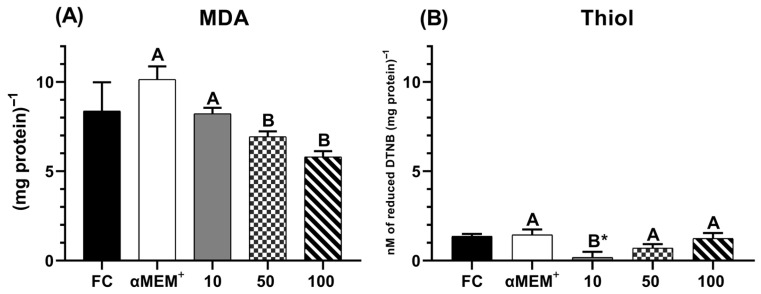
Levels of MDA (**A**) and Thiol (**B**) in bovine ovarian tissue before (fresh control, FC) and after 6 days of in vitro culture in the absence (αMEM^+^) or presence of ethanolic extract from *Punica granatum* L. (EE-PG) at 10, 50 and 100 µg/mL. ^A,B^ Different capital letters denote significant differences among treatments at day 6 of in vitro culture (*p* < 0.05). * Indicates difference from FC (*p* < 0.05). The analyses were performed using one-way ANOVA followed by Tukey’s post hoc test.

Figure 7 displays the levels of malondialdehyde (MDA) and thiol in bovine ovarian tissue cultured for 6 days. The intermediate and highest concentrations of EE-PG demonstrated a better protective effect against lipid peroxidation (*p* = 0.0376 and *p* = 0.0031, respectively) (Figure 7A) compared to 10 µg/mL and the αMEM^+^ alone (Culture control). These concentrations also maintained thiol levels comparable to both the cultured and fresh (D0) controls (Figure 7B).

## 4. Discussion

This study is the first to demonstrate the effects of the ethanolic extract of *Punica granatum* (EE-PG) on the in vitro culture of preantral follicles enclosed in bovine ovarian tissue. EE-PG enhanced redox balance by increasing its antioxidant capacity in a concentration-dependent manner, maintaining GSH levels and reducing MDA levels, preserving follicular morphology and ultrastructure, and supporting a high type I/type III collagen ratio. These findings suggest that EE-PG not only aids follicular survival but also stimulates extracellular matrix remodeling in cultured ovarian stroma. Similar protective effects of natural antioxidants on in vitro ovarian cultures have been reported for melatonin and resveratrol, reinforcing the relevance of EE-PG as an alternative [4,10].

Punicalagin is a major phenolic compound constituting approximately 70% of the total polyphenols in pomegranate peel [31]. In our chromatographic analyses, we confirmed the presence of α-punicalagin in the EE-PG, as previously shown [32]. Punicalagin has been widely recognized for its antioxidant properties [16,33]. It has these properties due to its ability to act as a potent electron donor through its hydroxyl functional groups, thereby neutralizing free radicals [34]. Therefore, we believe that the antioxidant capacity of the EE-PG observed in the present study is likely due to its phenolic compounds, particularly punicalagin. This is in agreement with reports showing punicalagin-mediated protection against oxidative stress in different models, including reproductive tissues [6,8,11]. Cervantes-Anaya et al. [35] demonstrated in their studies that the antioxidant effect of an aqueous pomegranate extract was superior when compared to pure punicalagin and ellagic acid, which are major components commonly found in extracts produced from this fruit. However, it is still not well understood how the possible synergistic effects among the components present in the extract occur.

Additionally, EE-PG mitigated lipid peroxidation by reducing MDA levels, a mechanism that is supported by findings in diabetic rats [36]. The elevated thiol levels that were observed further corroborate the antioxidative potential of EE-PG, emphasizing its role in maintaining a less oxidative environment. The minimal oxidative stress likely prevented significant activation of antioxidant enzymes, such as SOD and CAT. One plausible explanation is that EE-PG prevented excessive ROS formation, thereby reducing the need for enzymatic defenses. However, alternative mechanisms must be considered: phenolic compounds, including punicalagin, may directly modulate antioxidant enzymes at the transcriptional or post-translational level [12] Such nuances strengthen the need for molecular studies to clarify whether the effect of EE-PG is limited to ROS scavenging or extends to regulation of enzymatic pathways.

Furthermore, the preservation of follicular structure, follicular development, and the maintenance of type I collagen proportions underscore the potential of EE-PG to support ovarian tissue integrity during in vitro culture. This result aligns with findings that type I collagen is a key ECM component during folliculogenesis, while type III is associated with tissue remodeling [25,37]. These results may be mediated by the regulatory effects of punicalagin on extracellular matrix remodeling [11,33,34].

Another important aspect observed was that the percentage of type I collagen fibers remained elevated, while the percentage of type III collagen was reduced, resulting in a higher type I/type III collagen ratio. Collagen is one of the major components of the ovarian extracellular matrix (ECM), with type I collagen being the most prevalent throughout the organism. Furthermore, type I collagen is frequently found in association with type III collagen, contributing to the formation and maintenance of the ovarian stroma. Type I collagen plays a crucial role in the mammalian ovary, such as during luteolysis, in which its expression increases [37]. Thus, the higher ratio of type I/III collagen induced by EE-PG may favor structural stability and promote adequate cell–cell communication in the ovarian cortex.

In addition, the culture of follicles enclosed in the ovarian cortex, also known as an in situ culture system, is considered comparable to the in vivo ovarian environment. In situ culture provides a more complex support system [38], delivering local biochemical signals that promote follicular development, such as activation of the PI3K/Akt pathway. These conditions preserve follicular integrity and maintain contact with surrounding stromal cells, reducing the probability of necrosis, by increasing the contact area to facilitate nutrient exchange, ensuring follicular growth [38]. Although no influence of EE-PG on stromal cell density was observed, we hypothesize that the maintenance of ultrastructural integrity may still stimulate the remodeling processes of bovine ovarian stroma, thereby supporting follicular development. This hypothesis requires further studies evaluating cell–ECM interactions under EE-PG treatment. In addition, Kolesarova et al. [39] demonstrated that pomegranate peel extract can modulate steroidogenesis in HGL5 granulosa cells by enhancing 17β-estradiol secretion. This finding suggests that the action of pomegranate may extend specifically to follicular somatic cells, highlighting a distinct cellular target compared to stromal cells, which were the focus of evaluation in the present study.

Regarding the redox equilibrium, the interaction of free radicals with free or membrane lipids gives rise to lipid peroxidation, a process that can harm mammalian folliculogenesis. In this process, unsaturated fatty acids interact with hydroxyl radicals (HO•) to produce MDA. MDA, in turn, is a molecule that may cause damage to cell structure and is capable of binding to proteins and DNA [3]. Consequently, some researchers have demonstrated that the antioxidant properties of pomegranate are attributable to its capacity to reduce MDA levels [34]. These findings corroborate the results from our study, since EE-PG decreased MDA levels in ovarian tissue after in vitro culture, preventing lipid peroxidation and protecting the tissue from damage caused by free radicals. Moreover, in the present study, EE-PG was capable of maintaining high thiol levels, which ratify its antioxidant potential. Unlike MDA, thiol levels increase in a less oxidant environment, as we have previously shown [27]. Therefore, we can hypothesize that EE-PG prevents oxidative stress in the in vitro environment, thereby maintaining redox equilibrium. Together, these effects point to EE-PG as a promising candidate for supporting ovarian tissue culture and fertility preservation.

Given that ovarian tissue was maintained in an in vitro environment that was less oxidative, antioxidant defenses did not need to be activated. This can be explained by the low activity of SOD and CAT enzymes. SOD converts superoxide anion (O_2_^−•^) into hydrogen peroxide (H_2_O_2_), and CAT, consequently, converts H_2_O_2_ into water [3]. It is plausible that EE-PG, by hindering the generation of free radicals, impeded the recruitment of this antioxidant defense. However, considering previous studies with melatonin and punicalagin in bovine ovarian tissues [11,18], it is equally possible that EE-PG modulates signaling cascades, such as Nrf2, that regulate antioxidant enzymes.

From a translational standpoint, these findings extend beyond fundamental ovarian physiology. Natural antioxidants such as EE-PG represent promising agents for use in assisted reproduction and fertility preservation programs, applicable to both livestock production and clinical settings for women undergoing chemotherapy [10]. Future studies are needed to ascertain whether EE-PG can enhance oocyte maturation and developmental competence, as observed with other antioxidants, and whether its efficacy can be optimized via alternative extraction methods or combined supplementation strategies.

While the functional parameters assessed herein provide an essential foundation, there remains ample scope for expansion to comprehensively characterize the reproductive potential, given that direct endpoints (e.g., oocyte maturation, fertilization, and embryonic development) were not the primary focus of this evaluation. Moreover, although various EE-PG concentrations were tested, the optimization of the effective dose—and the investigation of potential synergistic effects with other antioxidants—constitutes a critical direction for future research. Finally, while the exploration of molecular mechanisms has been initiated, it necessitates further, dedicated inquiry. Detailed analyses of gene and protein expression related to antioxidant enzymes and pivotal signaling pathways, such as Nrf2, are imperative to solidify the understanding of EE-PG’s effects.

## 5. Conclusions

Our study demonstrated that the ethanolic extract from pomegranate peel (EE-PG) contributed to the preservation of the morphology and ultrastructure of preantral follicles, as well as to the maintenance of redox balance in bovine ovarian tissue cultured in vitro. These findings highlight the innovative potential of EE-PG as a natural, accessible, and promising supplement for in vitro follicular preservation strategies. Nevertheless, complementary studies could be conducted, such as the analysis of gene and protein expression related to follicle maturation, survival, and apoptosis, as well as other oxidative stress markers. In addition, comparative studies using different extraction methods and concentrations of the extract could help determine the optimal efficacy of EE-PG on folliculogenesis in bovines and other species.

## Data Availability

The original contributions presented in this study are included in the article. Further inquiries can be directed to the corresponding author.

## References

[1] Luz H.K.M., Wanderley L.S., Faustino L.R., da Silva C.M.G., de Figueiredo J.R., Rodrigues A.P.R. (2011). Papel de agentes antioxidantes na criopreservação de células germinativas e embriões. Acta Sci. Vet..

[2] Silva B.R., Silva J.R.V. (2023). Mechanisms of action of non-enzymatic antioxidants to control oxidative stress during in vitro follicle growth, oocyte maturation, and embryo development. Anim. Reprod. Sci..

[3] das Chagas Costa F., Vasconcelos E.M., Silva J.R.V., Liza A., Batista P.S. (2022). Influência das espécies reativas de oxigênio durante o cultivo in vitro de oócitos e folículos ovarianos de mamíferos domésticos. Rev. Bras. Reprodução Anim..

[4] Silva B.R., Costa F.C., Neto M.F.D.L., Filho F.F.C., de Assis E.I., Aguiar F.L., Silva A.W., Martins S.D., Araújo V.R., Matos M.H. (2024). Melatonin acts through different mechanisms to control oxidative stress and primordial follicle activation and survival during in vitro culture of bovine ovarian tissue. Domest. Anim. Endocrinol..

[5] De Ligny W., Smits R.M., Mackenzie-Proctor R., Jordan V., Fleischer K., De Bruin J.P., Showell M.G. (2022). Antioxidants for male subfertility. Cochrane Database Syst. Rev..

[6] Kulkarnia A.P., Aradhyaa S.M., Divakarb S. (2004). Isolation and identification of a radical scavenging antioxidant–Punicalagin from pith and carpellary membrane of pomegranate fruit. Food Chem..

[7] Seeram N., Adams L., Henning S., Niu Y., Zhang Y., Nair M., Heber D. (2005). In vitro antiproliferative, apoptotic and antioxidant activities of punicalagin, ellagic acid and a total pomegranate tannin extract are enhanced in combination with other polyphenols as found in pomegranate juice. J. Nutr. Biochem..

[8] Sun W., Yan C., Frost B., Wang X., Hou C., Zeng M., Gao H., Kang Y., Liu J. (2016). Pomegranate extract decreases oxidative stress and alleviates mitochondrial impairment by activating AMPK-Nrf2 in hypothalamic paraventricular nucleus of spontaneously hypertensive rats. Sci. Rep..

[9] Khalaf H.A., Arafat E.A., Ghoneim F.M. (2019). A histological, immunohistochemical and biochemical study of the effects of pomegranate peel extracts on gibberellic acid induced oxidative stress in adult rat testes. Biotech. Histochem..

[10] Liu H., Zeng Z., Wang S., Li T., Mastriani E., Li Q.-H., Bao H.-X., Zhou Y.-J., Wang X., Liu Y. (2017). Main components of pomegranate, ellagic acid and luteolin, inhibit metastasis of ovarian cancer by down-regulating MMP2 and MMP9. Cancer Biol. Ther..

[11] Bellusci G., Mattiello L., Iannizzotto V., Ciccone S., Maiani E., Villani V., Diederich M., Gonfloni S. (2019). Kinase-independent inhibition of cyclophosphamide-induced pathways protects the ovarian reserve and prolongs fertility. Cell Death Dis..

[12] Bezerra V.S., Costa F.C., Filho F.F.C., Costa J.J.N., Neto M.F.d.L., Furtado C.L.M., Ceccatto V.M., Araújo V.R., Silva J.R.V. (2024). Punicalagin increases follicular activation, development and activity of superoxide dismutase 1, catalase, and glutathione peroxidase 1 in cultured bovine ovarian tissues. Reprod. Fertil. Dev..

[13] Ahmed S.T., Yang C. (2017). Effects of dietary *Punica granatum* L. by-products on performance, immunity, intestinal and fecal microbiology, and odorous gas emissions from excreta in broilers. J. Poult. Sci..

[14] Saeed M., Naveed M., BiBi J., Kamboh A.A., Arain M.A., Shah Q.A., Alagawany M., El-Hack M.E.A., Abdel-Latif M.A., Yatoo M.I. (2018). The promising pharmacological effects and therapeutic/medicinal applications of *Punica granatum* L. (Pomegranate) as a functional food in humans and animals. Pat. Inflamm. Allergy Drug Discov..

[15] Yayla M., Cetin D., Adali Y., Kilicle P.A., Toktay E. (2018). Potential therapeutic effect of pomegranate seed oil on ovarian ischemia/reperfusion injury in rats. Iran. J. Basic Med. Sci..

[16] Nascimento J.E.T.D., Rodrigues A.L.M., De Lisboa D.S., Liberato H.R., Falcão M.J.C., Da Silva C.R., Júnior H.V.N., Filho R.B., Junior V.F.D.P., Alves D.R. (2018). Chemical Composition and Antifungal In Vitro and In Silico, Antioxidant, and Anticholinesterase Activities of Extracts and Constituents of *Ouratea fieldingiana* (DC.) Baill. Evid. Based Complement. Altern. Med..

[17] Çam M., Hısıl M. (2010). Pressurised water extraction of polyphenols from pomegranate peels. Food Chem..

[18] Jimenez C.R., Araújo V.R., Penitente-Filho J.M., de Azevedo J.L., Silveira R.G., Torres C.A.A. (2016). The base medium affects ultrastructure and survival of bovine preantral follicles cultured in vitro. Theriogenology.

[19] Silva J.R.V., Van den Hurk R., Figueiredo J.R. (2004). In vitro culture of preantral follicles: Advances and perspectives. Anim. Reprod. Sci..

[20] Magalhães-Padilha D.M., Andrade E.R., Sales A.D., Rodrigues G.Q., Lima L.F., Lunardi F.O., Apolloni L.B., Peixoto C.A., Silva J.R.V. (2012). In vitro survival and development of goat preantral follicles cultured in two-dimensional and three-dimensional systems using α-MEM or TCM-199 media. Theriogenology.

[21] Morais A., Lima L., Silva A., Lienou L., Ferreira A., Watanabe Y., Joaquim D., Alves B., Pereira A., Alves D. (2023). Effect of carvacrol antioxidant capacity on oocyte maturation and embryo production in cattle. Zygote.

[22] Araújo V.R., Gastal M.O., Figueiredo J.R., Gastal E.L. (2014). In vitro culture of bovine preantral follicles: A review. Reprod. Biol. Endocrinol..

[23] Alves K.A., Alves B.G., Gastal G.D.A., de Tarso S.G.S., Gastal M.O., Figueiredo J.R., Gambarini M.L., Gastal E.L. (2016). The Mare Model to Study the Effects of Ovarian Dynamics on Preantral Follicle Features. PLoS ONE.

[24] Rittíe L. (2017). Fibrosis.

[25] Junqueira L.C.U., Cossermelli W., Brentani R. (1978). Differential staining of collagens type I, II and III by Sirius red and polarization microscopy. Arch. Histol. Jpn..

[26] Takahashi H., Nara Y., Tuzimura K. (1978). Fluorometric determination of glutathione by N-(9-acridinyl)maleimide and its application to biological materials. Agric. Biol. Chem..

[27] Cighetti G., Debiasi S., Paroni R., Allevi P. (1999). Free and total malondialdehyde assessment in biological matrices by gas chromatography–mass spectrometry: What is needed for an accurate detection. Anal. Biochem..

[28] Furtado R.L., Martins J.E.R., Oliveira M.A.F., Guerreiro D.D., de Sá N.A.R., Ferraz A.S.M., Ceccatto V.M., Rodrigues A.P.R., Araújo V.R. (2021). Acute effect of high-intensity interval training exercise on redox status in the ovaries of rats fed a high-fat diet. Reprod. Fertil. Dev..

[29] Bannister J.V., Calabrese L. (1987). Assays for superoxide dismutase. Methods Biochem. Anal..

[30] Aebi H. (1984). Catalase in vitro. Methods Enzymol..

[31] Barbieri M., Heard C.M. (2019). Isolation of punicalagin from *Punica granatum* rind extract using mass-directed semi-preparative ESI-AP single quadrupole LC-MS. J. Pharm. Biomed. Anal..

[32] Lu J., Wei Y., Yuan Q. (2007). Preparative separation of punicalagin from pomegranate husk by high-speed countercurrent chromatography. J. Chromatogr. B.

[33] Talekar S., Patti A.F., Vijayraghavan R., Arora A. (2019). Rapid, enhanced and eco-friendly recovery of punicalagin from fresh waste pomegranate peels via aqueous ball milling. J. Clean. Prod..

[34] Ramlagan P., Labib R.M., Farag M.A., Neergheen V.S. (2022). Advances towards the analysis, metabolism and health benefits of punicalagin, one of the largest ellagitannins from plants, with future perspectives. Phytomed. Plus..

[35] Cervantes-Anaya N., Azpilcueta-Morales G., Estrada-Camarena E., Ortega D.R., de la Cruz V.P., González-Trujano M.E., López-Rubalcava C. (2022). Pomegranate and Its Components, Punicalagin and Ellagic Acid, Promote Antidepressant, Antioxidant, and Free Radical-Scavenging Activity in Ovariectomized Rats. Front. Behav. Neurosci..

[36] Aboonabi A., Rahmat A., Othman F. (2014). Antioxidant effect of pomegranate against streptozotocin-nicotinamide generated oxidative stress induced diabetic rats. Toxicol. Rep..

[37] Parkes W.S., Amargant F., Zhou L.T., Villanueva C.E., Duncan F.E., Pritchard M.T. (2021). Hyaluronan and collagen are prominent extracellular matrix components in bovine and porcine ovaries. Genes.

[38] Picton H.M., Harris S.E., Muruvi W., Chambers E.L. (2008). The in vitro growth and maturation of follicles. Reproduction.

[39] Kolesarova A., Baldovska S., Kohut L., Vasicek J., Ivanisova E., Arvay J., Duracka M., Roychoudhury S. (2023). Modulatory effect of pomegranate peel extract on key regulators of ovarian cellular processes in vitro. Front. Endocrinol..

